# Supplementary material for: Effects of Edible Insect Tenebrio molitor Larva Fermentation Extract as a Substitute Protein on Hepatosteatogenesis and Proteomic Changes in Obese Mice Induced by High-Fat Diet
Source: Int J Mol Sci. 2021 Mar 31;22(7):3615. doi: 10.3390/ijms22073615 (PMC8037111; doi:10.3390/ijms22073615)
Supplement: Supplementary file 1 [file ijms-22-03615-s001.pdf]

Table S1. Primer sequences.

Genes	Primer sequences (5'-3')
ADRP	TGGCTGTAAACGTCTGTCTGG/GCACACGCCTTGAGAGAAAC
CD36	GCTGTCAGGCGTCAGGATAA/TGGCTTCAGGGAGACTGTTG
ChREBP	CTGGGGACCTAAACAGGAGC/GAAGCCACCCTATAGCTCCC
DGAT2	CTGGCTGATAGCTGCTCTCTACTTG/TGTGATCTCCTGCCACCTTTC
FAS	TTGGAGCTAAGGCATGGTGG/GCAGTTGTCCTCTGGATGCT
GAPDH	AAGGTCATCCCAGAGCTGAA/CTGCTTCACCACCTTCTTGA
PAP	GGGTTCTACTGTGGAGATGA/TGACAGTAGCTGTGATGATGA
PPARγ	TCGCTGATGCACTGCCTATG/GAGAGGTCCACAGAGCTGAT
SREBP-1c	AACCTCATCCGCCACCTG/TGGTAGACAACAGCCGCATC
